# Mosquito densovirus significantly reduces the vector susceptibility to dengue virus serotype 2 in *Aedes albopictus* mosquitoes (Diptera: Culicidae)

**DOI:** 10.1186/s40249-023-01099-8

**Published:** 2023-05-09

**Authors:** Ling Kong, Jie Xiao, Lu Yang, Yuan Sui, Duoquan Wang, Shaoqiang Chen, Peiwen Liu, Xiao-Guang Chen, Jinbao Gu

**Affiliations:** 1 Guangdong Provincial Key Laboratory of Tropical Disease Research, Department of Pathogen Biology, School of Public Health, Southern Medical University, Guangzhou, 510515 Guangdong China; 2 Brown School, Washington University, St. Louis, MO 63130 USA; 3 National Institute of Parasitic Diseases, Chinese Center for Disease Control and Prevention, Shanghai, 200025 China; 4 Shenzhen Aiming Pest Control Operation Service Company Limited, Shenzhen, Guangdong China

**Keywords:** DENV-2, Mosquito densovirus, Superinfection, *Aedes albopictus*, Vector susceptibility

## Abstract

**Background:**

Dengue virus (DENV) is a major public health threat, with *Aedes albopictus* being the confirmed vector responsible for dengue epidemics in Guangzhou, China. Mosquito densoviruses (MDVs) are pathogenic mosquito-specific viruses, and a novel MDV was previously isolated from *Ae. albopictus* in Guangzhou. This study aims to determine the prevalence of MDVs in wild *Ae. albopictus* populations and investigate their potential interactions with DENV and impact on vector susceptibility for DENV.

**Methods:**

The prevalence of MDV in wild mosquitoes in China was investigated using open access sequencing data and PCR detection in *Ae. albopictus* in Guangzhou. The viral infection rate and titers in MDV-persistent C6/36 cells were evaluated at 12, 24, 48, 72, 96, and 120 h post infection (hpi) by indirect immunofluorescence assay (IFA) and real time quantitative PCR (RT-qPCR). The midgut infection rate (MIR), dissemination rate (DR), and salivary gland infection rate (SGIR) in various tissues of MDV-infected mosquitoes were detected and quantified at 0, 5, 10, and 15 days post infection (dpi) by RT-PCR and RT-qPCR. The chi-square test evaluated dengue virus serotype 2 (DENV-2) and *Aedes aegypti* densovirus (AaeDV) infection rates and related indices in mosquitoes, while Tukey's LSD and *t*-tests compared viral titers in C6/36 cells and tissues over time.

**Results:**

The results revealed a relatively wide distribution of MDVs in *Aedes*, *Culex*, and *Anopheles* mosquitoes in China and an over 68% positive rate. In vitro*,* significant reductions in DENV-2 titers in supernatant at 120 hpi, and an apparent decrease in DENV-2-positive cells at 96 and 120 hpi were observed. In vivo, DENV-2 in the ovaries and salivary glands was first detected at 10 dpi in both monoinfected and superinfected *Ae. albopictus* females, while MDV superinfection with DENV-2 suppressed the salivary gland infection rate at 15 dpi. DENV-2 titer in the ovary and salivary glands of *Ae. albopictus* was reduced in superinfected mosquitoes at 15 dpi.

**Conclusions:**

MDVs is widespread in natural mosquito populations, and replication of DENV-2 is suppressed in MDV-infected *Ae. albopictus*, thus reducing vector susceptibility to DENV-2. Our study supports the hypothesis that MDVs may contribute to reducing transmission of DENV and provides an alternative strategy for mosquito-transmitted disease control.

**Graphical abstract:**

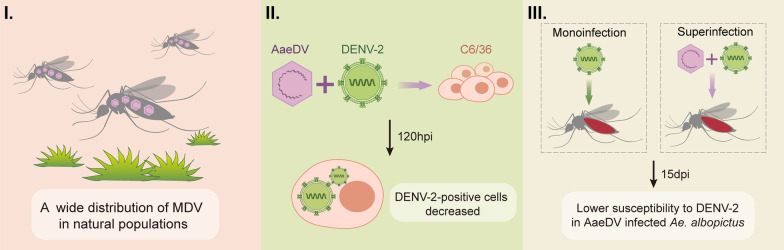

**Supplementary Information:**

The online version contains supplementary material available at 10.1186/s40249-023-01099-8.

## Background

Mosquito densoviruses (MDVs) are mosquito specific, entomopathogenic icosashedral, nonenveloped viruses 20–25 nm in diameter, with a single-stranded linear DNA genome ranging from 4 to 6 kilobases that ends in two hairpin structures [1]. According to a recent taxonomy revision, MDVs belong to the *Parvoviridae* family, the *Hamaparvovirinae* subfamily, and the *Brevihamaparvovirus* genus [2, 3].

In 1972, the first MDV, *Aedes aegypti* densovirus (AaeDV), was identified in an *Ae. aegypti* laboratory strain in Russia [4]. MDVs have been continuously isolated from mosquito cell lines maintained in laboratories, established mosquito laboratory colonies, and wild populations of mosquitoes [5]. Most MDVs show variable degrees of pathogenicity in their mosquito host [6]. MDVs can invade and proliferate in multiple organs and tissues of mosquitoes, and infection with MDVs can cause larval death or deformation. MDVs extend the larval stage and decrease the lifespan and body size of adults [1]. Furthermore, their pathogenicity can be significantly improved by genetic engineering techniques, such as altering the vector used to express the insect-specific toxin [7], gene or specific short hairpin RNA or artificial micro RNA that targets genes essential for the development, growth, or physiology of mosquitoes [8, 9]. MDVs can be transmitted in mosquito populations through horizontal transmission in larval habitats or vertical transmission when surviving infected females transmit the virus to offspring or through venereal transmission during mating [1, 10, 11]. Overall, these biological and pathogenic characteristics confer MDVs with potential as biological control agents [5].

A few surveys have revealed that MDVs are more likely widespread in natural populations, such as in *Ae. aegypti*, *Anopheles minimus* and *Culex pipiens* [23, 24]. For example, the overall MDV positive rate reached 18.8% in wild larvae and 15% in adults of *An. minimus* collected from Ban Phu Toei, a village in Thailand, when using PCR-based detection methods [12]. Another PCR survey of the MDV infection rate in adult *Ae. aegypti* and *Ae. albopictus* also carried out in Thailand showed that only the *Ae. aegypti* wild population was infected with MDV, with an overall prevalence of 44% [13]. A more large-scale survey of the general prevalence of *Culex pipiens* densovirus (CpDV) in natural *Cx. pipiens* (s.l.) populations was recently reported; more than two thousand *Cx. pipiens* were collected from 136 different locations worldwide, and 48% of the samples were CpDV-positive, as determined by PCR diagnostic tests [14].

Dengue virus (DENV) belongs to the family *Flaviviridae*, genus *Flavivirus*, and is primarily transmitted to humans through the bite of infected adult female mosquitoes, notably *Ae. aegypti* and *Ae. albopictus* [15, 16]. Guangdong Province, which is located in southeast China, is a hyperendemic region of dengue transmission. Since the first outbreak of dengue fever in 1978, over 0.72 million cumulative cases have been recorded within the last 40 years, accounting for approximately 90 percent of cases in China [17]. Since 2010, dengue virus serotype 1 (DENV-1) and dengue virus serotype 2 (DENV-2) have been the predominant serotypes in Guangdong. However, current epidemiological analysis supports that dengue has not yet developed into an endemic arboviral disease in Guangdong, with local dengue outbreaks still being initiated by imported cases [17, 18]. *Ae. albopictus* is the only confirmed transmission vector responsible for these dengue epidemics in Guangdong [17].

Previously, we isolated and characterized novel *Aedes albopictus* densovirus-7 (AalDV-7) from wild-caught *Ae. albopictus* in the dengue-endemic area of Guangzhou City, Guangdong Province [6], leading to questions about whether MDV widely exists in the wild population of *Ae. albopictus* and what their potential influence on DENV transmission by mosquitoes might be. In this study, we first used DENV-2 to superinfect C6/36 cells persistently infected with AaeDV and determined the impact of such superinfection on DENV-2 by examining persistent infection. To further explore a competitive interference in vivo, we also assessed whether the presence of AaeDV in live mosquitoes interferes with the vector susceptibility to DENV-2 and sequential infection modes based on the likelihood that sequential challenges are most frequent in nature.

## Methods

### Identification of MDV presence using open access sequencing data

Metagenomic next-generation sequencing (NGS) data for field mosquitoes in China were searched from the NCBI SRA database (https://www.ncbi.nlm.nih.gov/sra) and CNGB Sequence Archive database (https://db.cngb.org/cnsa/) using the keywords “mosquito OR *Anopheles* OR *Aedes* OR *Culex*”. Followed by removing the datasets with less than 1 million reads and keeping those derived from the field in China, a total of 29 published datasets from five provincial level administrative divisions (PLADs) were retrieved, including four from Guangdong (SRP188743), three from Yunnan (SRP148705), eleven from Ningxia, five from Gansu, and six from Shanxi (CNP0001261) (details in Additional file 1: Table S1).

Twenty-six mosquito densovirus (MDV) genomes were downloaded as the reference from GenBank using the search keywords “densovirus” AND “(mosquito OR *aAnopheles* OR *aAedes* OR *Culex*)”, with sequence length limited to longer than 3500 bp (see Additional file 2: Table S2 for the MDV accession number).

Adaptor and low-quality bases were removed with TrimGalore (v0.6.7) [19]. The remaining clean reads were aligned to the genomes of *Ae. albopictus* (AaloF1.2, GCA_001444175.1), *Cx. quinquefasciatus* (JHB2020, GCA_015732765.1)*,* and *An. sinensis* (AsinS2.6, GCA_000472065.2) by bowtie2 (v2.4.4) [20]*.* All these genomes were downloaded from Vectorbase (http://www.vectorbase.org). Reads mapping to host genomes were removed to exclude interference from the host genome and possible insertion of viral sequences, and unmapped reads were aligned to the MDV reference using blatsn (v2.4.4, NIH, USA) with the parameter “evalue < 1e−10”. The mapped reads were retained as MDV-related reads. The abundance of MDV reads was divided by the library counts and multiplied by a million normalized to CPM.

### Mosquito collection

**Fig. 1:**
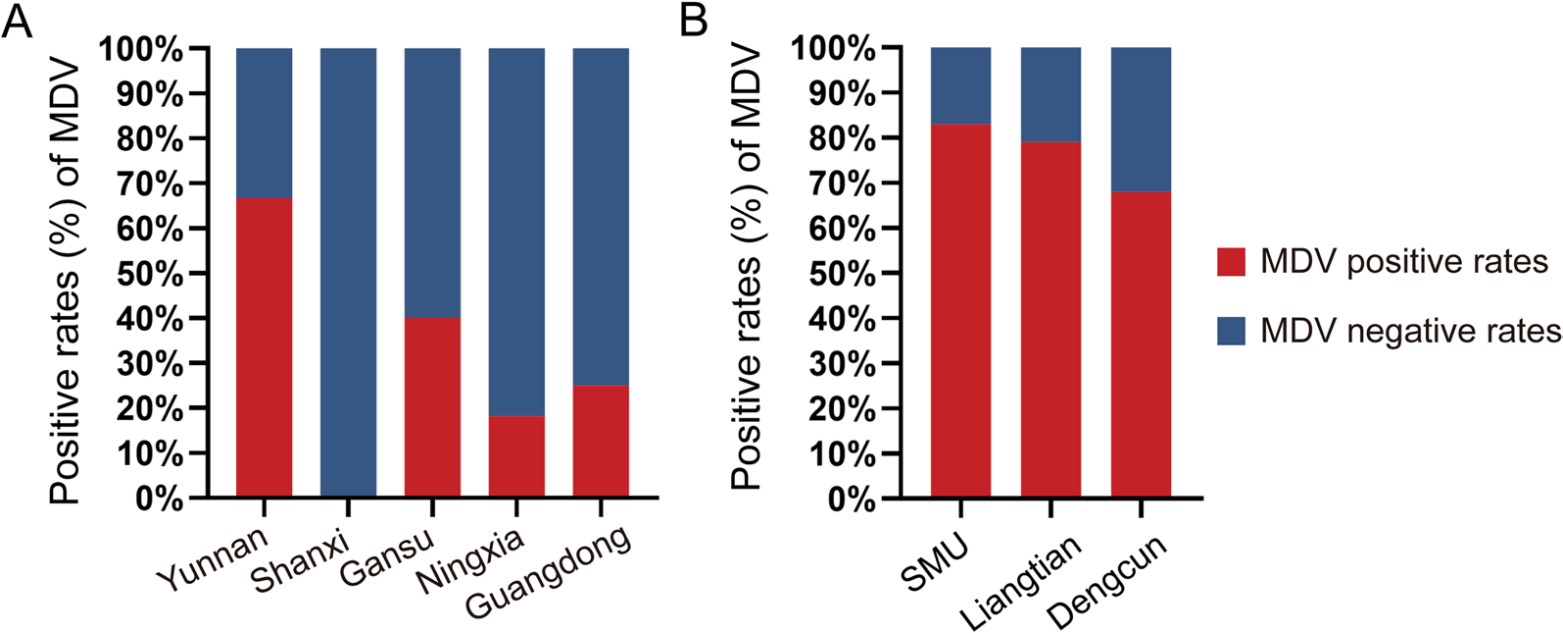
Analysis of mosquito densovirus (MDV) positive rates in natural mosquito populations. **A** Stacked column chart showing the mosquito densovirus positive rates (red) and negative rates (blue) of reads in field mosquito metagenomic data from five provincial level administrative divisions. **B** MDV positive rates (red) and negative rates (blue) in natural mosquito populations collected from three sites in Guangzhou. *SMU* The residential district of Southern medical university

Guangzhou is the third largest city in China and the largest in the southern part of the country. The population of Guangzhou is estimated to be over 13,900,000 in 2022. The region around Guangzhou is known for a massive influx of migrants, with up to 30 million additional migrants living in the area for at least six months out of every year. The average annual temperature in Guangzhou is 22.4 °C, and the rainfall is approximately 2123 mm per year. Therefore, the environmental and social factors in Guangzhou facilitate DENV transmission, and DENV epidemics have been reported in this region for more than 40 years [21]. To investigate the general prevalence of MDVs in Guangzhou natural *Ae. albopictus* populations, three sites were selected for mosquito collections, the residential district of Southern Medical University (SMU) (113°19′E, 23°11′N, 31 m above sea level (a.s.l.), population density of > 3000 people/km^2^), Liangtian (113°23′E, 23°21′N, 25 m a.s.l.; population density of approximately 1000 people/km^2^), and Dengcun (113°339′E, 23°309′N, 42 m a.s.l., 100 people/km^2^), representing three different types of ecological habitats: urban, suburban and rural. Mosquitoes were collected between June and July 2022 from three sites in Guangzhou (Fig. 1): SMU, Dengcun, and Liangtian. The mosquitoes were collected by using human-baited double-net (HDN) traps. After placing on an ice bath, only *Ae. albopictus* female mosquitoes based on morphological characteristics were retained for further MDV infection detection.

### MDV presence in natural populations

DNA was extracted from individual female mosquitoes collected from natural populations from the 3 locations (*n* = 100 per site) and tested for MDV presence by traditional PCR (Additional file 3: Table S3). MDV-free samples from the laboratory colony were used as the negative control. A set of primers specific to MDVs was designed to amplify a 655 bp fragment (from position 899 to 1554 of the referenced AaeDV genome; GenBank accession number: M37899.1) located in the highly conserved NS1 open reading frame (ORF). This primer set was able to detect not only AaeDV, but also other related densoviruses, such as *Aedes albopictus* densovirus (AalDV), *Anopheles gambiae* densovirus (AgDV), *Culex pipiens* densovirus (CpDV), based on the high level of conservation in this region among MDVs (Fig. 1, Additional file 4: Fig. S1). Ribosomal protein S7 (*Rps7*) was used as an internal control and was amplified in the same test tube based on multiplex PCR (MPCR). See Additional file 3: Table S3 for primers and PCR protocols. All experiments were performed in a biosafety level 2 containment laboratory.

### Cells and mosquitoes

*Ae. albopictus* C6/36 cells (ATCC, Manassas, USA, Cat# CRL-1660, RRID: CVCL_Z230) were grown at 28 °C in Roswell Park Memorial Institute (RPMI) 1640 medium (Gibco BRL, NY, USA) supplemented with 10% fetal bovine serum (FBS; Gibco BRL, NY, USA) and maintained at 28 °C.

*Ae. albopictus* mosquitoes of Foshan strain were used in this study. The mosquitoes were reared under standard insectary conditions at a constant 28 ± 2 °C, 70–80% relative humidity, and a 12:12-h light/dark photoperiod. Larvae were reared in 26.5 cm by 15.5 cm stainless steel rectangular pans and fed commercial finely ground turtle food (INCH-GOLD, Shenzhen, China). Colony adults were maintained in stainless steel cages [20 (length) × 20 (width) × 30 (height) cm, volume = 12,000 cm^3^] and supplied with a cotton wick soaked in 10% sugar solution. Adult females were fed defibrinate sheep blood (Solarbio Life Sciences, Beijing, China) using Hemotek membrane feeding systems (Hemotek Ltd., Blackburn, UK) three and four days after emergence. Every batch of mosquitoes was examined by conventional PCR to ensure that the experimental mosquitoes were free of MDVs.

To establish the AaeDV persistently infected cell line, C6/36 cells were infected with AaeDV at a final concentration of 10^11^ copies/ml; the infected cells were serially passaged every two days after three days of inoculation with AaeDV. The persistently infected C6/36 cells were serially passaged ten times after inoculation with AaeDV and used for further study.

### Virus

pUCA is the infectious clone of AaeDV containing the full-length genomic DNA of AaeDV, which was kindly provided by Prof. Erica Suchman and Jonathan Carlson. The construction of plasmids has been previously described in detail [22]. The wildtype virus AaeDV was generated by transfecting pUCA into C6/36 cell lines according to a previously described method [22].

The dengue virus serotype 2 (DENV-2) New Guinea C strain (NGC) used throughout this study was maintained in the laboratory of the Guangdong Provincial Key Laboratory of Tropical Disease Research, School of Public Health, Southern Medical University. DENV-2 stocks were obtained by inoculating a nearly confluent monolayer of C6/36 cells in a 25 cm^2^ tissue culture flask, with the virus diluted to 1:6 in 3 ml RPMI-1640 medium containing 2% heat-inactivated fetal bovine serum (FBS; Gibco BRL, NY, USA). Supernatants from infected cells were collected three days after infection until an apparent cytopathic effect, centrifuged at 1500×*g* for 5 min, separated into 0.5 ml aliquots, and stored at − 80 °C. Viral titers are expressed as a 50% tissue culture infective dose (TCID_50_/ml), as based on the method described by Reed and Muench [23].

### AaeDV and DENV-2 quantification

The AaeDV genome copy number was quantified using real-time quantitative PCR (qPCR). In brief, nonencapsidated genomic DNA was removed by treatment with TURBO DNase (Ambion, Austin, TX, USA) at 37 °C for 1 h. Total encapsidated genomic DNA was extracted using a Viral DNA Kit (Omega Bio-Tek, GA, USA). A standard curve was constructed using serial tenfold dilutions of a linear plasmid at known concentrations. The details of these procedures were described previously [24]. Virus genome copy numbers were determined using a SYBR green-based qPCR assay with SuperReal qPCR PreMix (Tiangen Biotech, Beijing, China). The primer sequences and reaction conditions used were as described previously [24].

DENV-2 RNA was quantified by real-time RT-PCR (qRT-PCR) using previously described specific primers according to published reaction conditions [25]. A 1995-bp DNA fragment, amplified using DENV2-F/DENV2-R primers, was ligated into pMD18-T plasmid after linearization with *Eco*RI restriction enzyme (Thermo Fisher Scientific, MA, USA) (Additional file 3: Table S3). A standard curve for DENV-2 was generated using a serial tenfold dilution of the linear plasmid containing a fragment of DENV-2 spanning nucleotides 9937–10,113 according to published procedures [25]. Each sample was analyzed in triplicate. After the specificity of PCR products was checked by the melt curve, viral genome copy numbers were determined by absolute quantification based on cycle threshold (Ct) values and standard curves.

### Acute DENV-2 infection

AaeDV infected or uninfected C6/36 cells (10^6^) were preseeded in a 12-well plate 1 day before DENV-2 infection. The C6/36 cell monolayers were washed with PBS and infected with DENV-2 at an MOI of 1. The plates were rocked gently for 15 min at room temperature for adsorption and incubated at 37 °C for 2 h. Then, individual wells of C6/36 cells were washed three times with PBS, and fresh medium was added. The infection was proceeded at 28 ℃ for 12, 24, 48, 72, 96, and 120 h, and was confirmed by immunofluorescence assay and qRT-PCR.

### Antibodies and immunofluorescence assay

The details of the production of a rabbit polyclonal antibody against AaeDV NS1 have been described [6]. Mouse monoclonal anti-DENV virus E2 envelope glycoprotein antibodies were purchased from Abcam (Cambridge, MA, USA). The secondary antibodies used, donkey anti-rabbit IgG-Alexa Fluor-594 and goat anti-mouse IgG-Alexa Fluor-488, were purchased from Thermo Fisher Scientific (MA, USA).

C6/36 cells were grown on glass coverslips, washed with phosphate-buffered saline (PBS), fixed using an acetone/absolute alcohol (3:2) mixture, blocked with 1% BSA + 0.05% PBST solution for 2 h at 37 °C, and washed with PBST. Following incubation with the primary antibodies, the coverslips were washed with PBS and stained with Alexa Fluor 594-conjugated donkey anti-rabbit IgG and/or Alexa Fluor 488 Hoechst-conjugated goat anti-mouse stain for 1 h at room temperature. DAPI was used for nuclear staining. Images were visualized under a 100 × oil objective using a FluoView 1000 confocal microscope (Olympus, Tokyo, Japan). Analysis of images and calculation of positive signals of immunofluorescence were performed using ImageJ software (1.49v, NIH, USA) [26].

### Mosquito injections with AaeDV

Two-day-old *Ae. albopictus* female adults were infected with AaeDV via intrathoracic injection. Briefly, adults were anesthetized on ice, and approximately 69 nl of AaeDV stock (10^11^ GE/ml) was injected into the thorax under a microscope, as described previously [27, 28]. After injection, the adult mosquitoes were immediately transferred to small plastic cups (1000 ml, 11 cm diameter at the top), fed a 10% glucose solution through soaked cotton wicks and allowed to recover. Mosquitos were injected with RPMI 1640 medium only as mock-infected controls. Three independent biological replicates were included for each treatment (*n* = 30 per replicate).

### Superinfection interference in vitro and in vivo

To examine interference between DENV-2 and AaeDV in *Aedes* mosquito cells, stable and persistent AaeDV-infected C6/36 cells were superinfected with DENV at an MOI of 1. Genome copies of DENV-2 and AaeDV were measured in cell lysates and supernatant by qPCR, and the percentage of DENV-2- and AaeDV-positive/negative cells was calculated at 12, 24, 48, 72, 96, and 120 h post superinfection (hpi). To determine the effect of superinfection on the percentage of virus-positive C6/36 cells, we performed immunofluorescence microscopy in monoinfected and superinfected C6/36 cells, and quantified the fluorescence intensity using ImageJ software (1.49v, NIH, USA) [26].

Injected mosquitoes were allowed to recover under standard rearing conditions. Four days after injection, *Ae. albopictus* female mosquitoes were exposed for 2 h to infectious blood meals using a blood-soaked pledget technique in three independent replicates [29]. Each blood meal comprised fresh virus diluted in commercially available defibrinated sheep blood and 1% sucrose to provide a final viral titer of approximately 10^7^ TCID_50_/ml of DENV-2. After feeding, fully engorged females were selected and returned to standard rearing. To evaluate the influences of AaeDV infection on the susceptibility of *Ae. albopictus* to DENV infection, adult female mosquitoes were first infected with AaeDV [10^6^ genome equivalents (geq)/per mosquito] via thoracic injection and then artificially fed with a mixture of blood with approximately 10^9^ RNA copies/ml of DENV, as described in the methods section. The IR, DR, and SGIR were evaluated, and a monoinfected adult was used as a control. The workflow is shown in Fig. 5A and B.

### Vector susceptibility experiments

At 0, 5, 10, and 15 days post DENV-2 infection (dpi), surviving mosquitoes were anesthetized with CO_2_ and dissected on a precooled glass slide for vector susceptibility assays. Disposable insect microneedles were used to separate the midgut, ovaries, and salivary glands of each mosquito under a dissecting microscope. The samples were washed three times in PBS and then homogenized by using a Motor-Driven Tissue Grinder prior to TRIzol addition, and viral nucleic acid was extracted using MiniBEST Viral RNA/DNA Extraction Kit Ver. 5.0 (TaKaRa, Japan) following the manufacturer's protocol. First-strand cDNA synthesis and RT-PCR amplification were performed according to methods that have been described in detail previously [30]. The primer sequences and PCR conditions used are listed in Additional file 3: Table S3.

Vector susceptibility of *Ae. albopictus* to DENV-2 was evaluated by calculating the midgut infection rate (MIR; the number of infected midguts/the number of tested midguts), dissemination rate (DR; the number of infected ovaries/the number of infected midguts), and salivary gland infection rate (SGIR; the number of infected salivary glands/the number of tested mosquitoes) [31, 32]. Genome copies of AaeDV were quantified in all midguts, ovaries, and salivary gland and genome copies were further determined in DENV2-positive tissues by absolute qRT-PCR.

### Data analysis

In this study, logistic regression was used to examine the vector competence of *Ae. albopictus* for DENV-2. IR, DR, and SGIR were compared separately at different time points, and the *P value* was corrected by Bonferroni adjustments. Chi-square (and Fisher’s exact) tests were used to determine the difference in vector competence between the AaeDV-infected group and the negative group. Tukey's LSD tests of variance were performed post hoc on the DENV-2 levels in tissues for each group after log transformation. Student's *t* test was used at each time point to compare DENV-2 RNA copies between the AaeDV-infected and negative groups. A *P value* < 0.05 was considered statistically significant (*), with a *P value* < 0.01 representing strong statistical significance (**). Statistical analyses were conducted using SPSS 20.0 (IBM, Chicago, IL, United States).

## Results

### Prevalence analysis of MDV in natural mosquito populations from public databases

We characterized the prevalence of MDV in 30,034 mosquitoes in 29 pools, representing four mosquito species from five PLADs (Yunnan, Shaanxi, Gansu, Ningxia, and Guangdong). Following quality control, a total of 309,695,245 clean paired-end reads were retained, with an average of approximately 5.16 M reads per 500 mosquitoes (0.85–114.43 M reads per 500 mosquitoes), and used for the subsequent MDV identification analysis pipeline. Among them, 3,014,183 reads were identified as viral reads, accounting for 0.12% (355,113/309,695,245) of all clean reads and suggesting a rich diversity of MDVs harbored by mosquitoes. Excluding six libraries from Shanxi (no reads hits), the MDV positive rates of reads were 66.67% (2/3), 40.00% (2/5), 18.18% (2/11), and 25.00% (1/4) in Yunnan, Gansu, Ningxia, and Guangdong, respectively (Fig. 1, Additional file 4: Fig. S1). The CPM of MDVs ranged from 1.39 to 46,862.13 (Additional file 1: Table S1). The respective minimum infection rates in Shanxi, Yunnan, Gansu, Ningxia, and Guangdong were 0%, 0.03%, 0.05%, 0.02%, and 0.10% (Additional file 1: Table S1). The results suggest that the natural mosquito population commonly carries various amounts of MDVs.

DNA samples from a total of 300 individual mosquitoes collected between May and July 2022 from 3 different locations (SMU for urban, Dengcun for rural, and Liangtian for suburban) were subjected to a PCR-based diagnostic test, and a MDV-specific set of primers was designed to amplify 655 bp (from 899 to 1554) (Additional file 3: Table S3 for primers and PCR protocols, Fig. 1, Additional file 4: Fig. S1) located in the highly conserved NS1 ORF. As a result, 83.00%, 79.00%, and 68.00% of the samples were MDV-positive (Fig. 1) in the SMU, Liangtian, and Dengcun, respectively (Additional file 4: Fig. S1, Additional file 5: Fig. S2).

### Establishment of C6/36 cells persistently infected with MDV

**Fig. 2:**
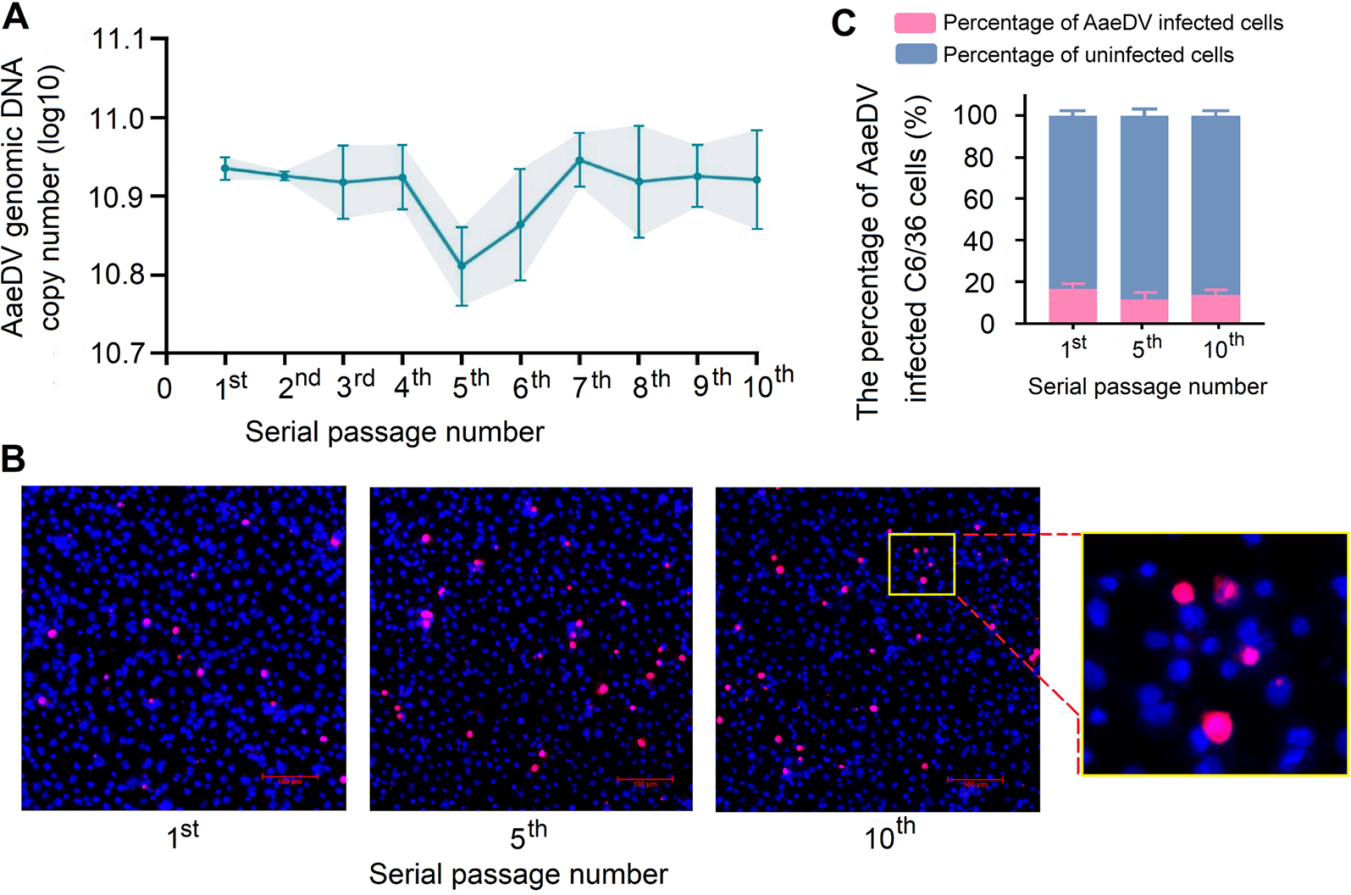
Determination of stable and persistent AaeDV infection in C6/36 cells. **A** Quantification of AaeDV genomic DNA from infected cells at the end of each passage up to the tenth passage. **B** Cells were fixed and permeabilized with an acetone/absolute alcohol mixture and probed with an anti-NS1 primary antibody and Alexa Fluor 594-conjugated goat anti-rabbit secondary antibody (red, infected cells). Cell nuclei were stained with DAPI (blue), and confocal images were acquired. The top-right insets show a zoomed image of cells harboring AaeDV, and the AaeDV NS1 protein is located predominantly in the nucleus of C6/36 cells. **C** The percentage of AaeDV-positive/negative cells was calculated by ImageJ software according to figure **B**. All results are expressed as the mean ± *SD*. *n* = 3

C6/36 cells were persistently infected with AaeDV without causing a discernible cytopathic effect (CPE) and grew normally compared with the uninfected C6/36 cells. The genome copy number of AaeDV was quantified at the end of each passage using a quantitative PCR assay. As shown in Fig. 2A, AaeDV DNA molecules from infected cells were maintained at relatively constant levels throughout ten passages. Furthermore, the total percentage of AaeDV-positive cells at the end of the first, fifth, and tenth passages was detected with an anti-NS1 antibody via IFA (Fig. 2B) The rates of infected C6/36 cells were sustained at approximately 16% (Fig. 2C). The above results indicate that stable and persistent AaeDV infection in C6/36 cells was established.

### DENV-2 superinfection in C6/36 cells persistently infected with AaeDV

**Fig. 3:**
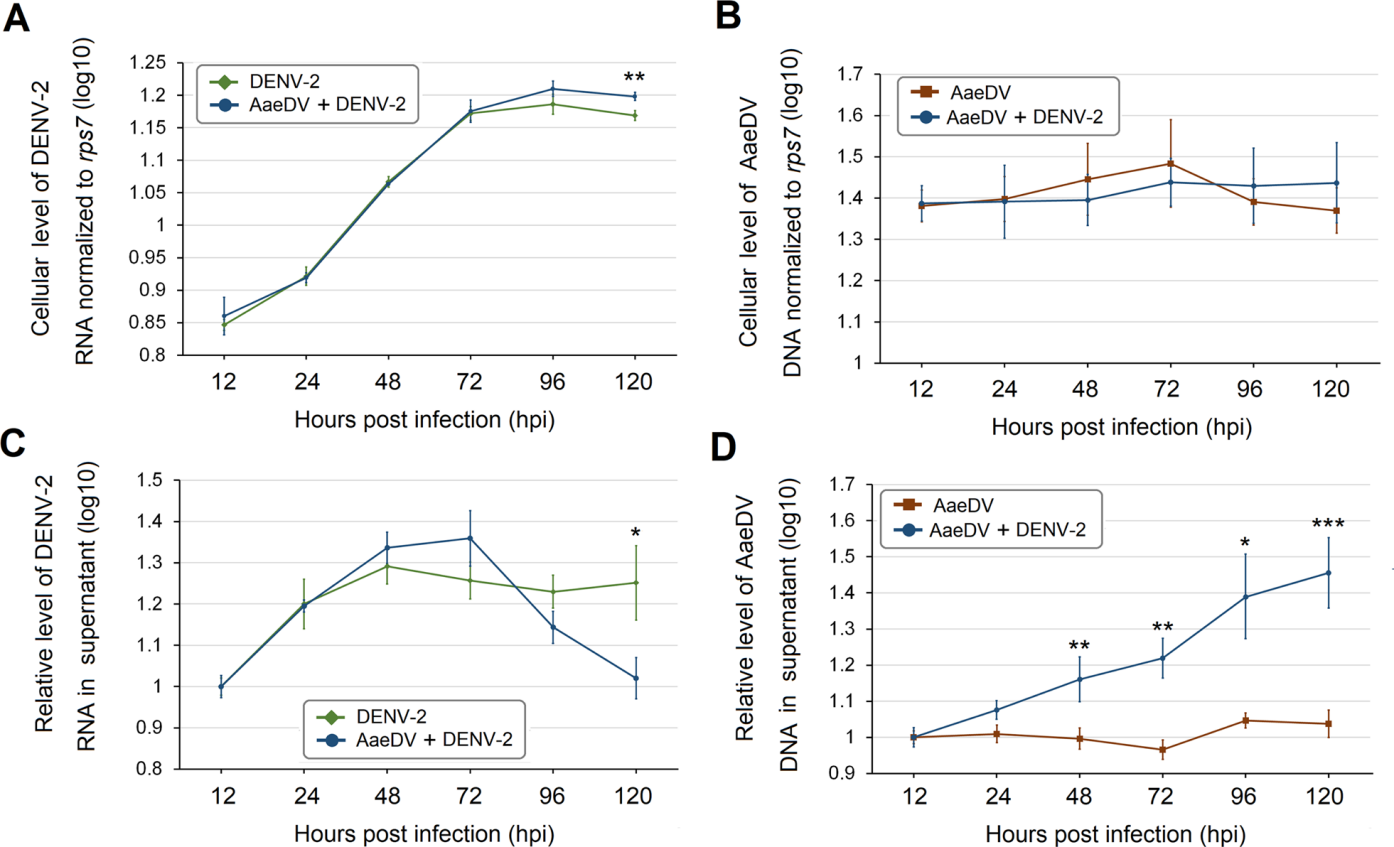
Growth characteristics of DENV-2 and AaeDV in monoinfection and superinfection treatments. RNA and DNA samples were prepared from cells and culture supernatant at different time points post infection, and qPCR assays were used to measure levels of DENV-2 RNA and AaeDV DNA. Intracellular levels of DENV-2 (**A**) and AaeDV (**B**) in monoinfection and superinfection treatments, respectively. Intracellular levels of DENV-2 and AaeDV were normalized to *rps7* mRNA in the same sample. Relative levels of DENV-2 (**C**) and AaeDV (**D**) in the supernatant of monoinfection and superinfection treatments. The relative level at 12 hpi was set as 1. The data presented are from a representative experiment that was repeated three times with similar results. Error bars represent the standard deviation

As shown in Fig. 3A, AaeDV-infected cell lysates were collected, followed by DENV-2 superinfection at 12, 24, 48, 72, and 96 hpi, with no significant difference in DENV-2 copies between the superinfected cells and single DENV-2-infected cells (12 hpi, *P* = 0.442; 24 hpi, *P* = 0.768; 48 hpi, *P* = 0.374; 72 hpi, *P* = 0.643; 96 hpi, *P* = 0.065). However, the DENV-2 RNA level increased slightly compared to single DENV-2 infection treatments at 120 hpi. Conversely, intracellular AaeDV replication in the superinfection treatment showed no significant difference from that in AaeDV monoinfection at any time point post superinfection (12 hpi, *P* = 0.855; 24 hpi, *P* = 0.880; 48 hpi, *P* = 0.460; 72 hpi, *P* = 0.538; 96 hpi, *P* = 0.540; 120 hpi, *P* = 0.371) (Fig. 3B).

In contrast, the dengue virus RNA copy number in the supernatant was suppressed at 120 h after DENV-2 superinfection compared with DENV-2 monoinfection (1.02 ± 0.05 and 1.25 ± 0.09). In contrast, the AaeDV genomic copy number was significantly enhanced 48 h after DENV-2 superinfection compared with AaeDV monoinfection, and the promotional effect of DENV-2 on AaeDV replication increased with time (48 hpi, 1.16 ± 0.06 and 1.00 ± 0.03; 72 hpi, 1.22 ± 0.05 and 0.97 ± 0.03; 96 hpi, 1.39 ± 0.10 and 1.05 ± 0.02; 120 hpi, 1.46 ± 0.09 and 1.04 ± 0.04). Overall, the relative level of AaeDV genome increased nearly 0.42-fold in the superinfection treatment compared with AaeDV monoinfection at 120 hpi (Fig. 3C and D).

**Fig. 4:**
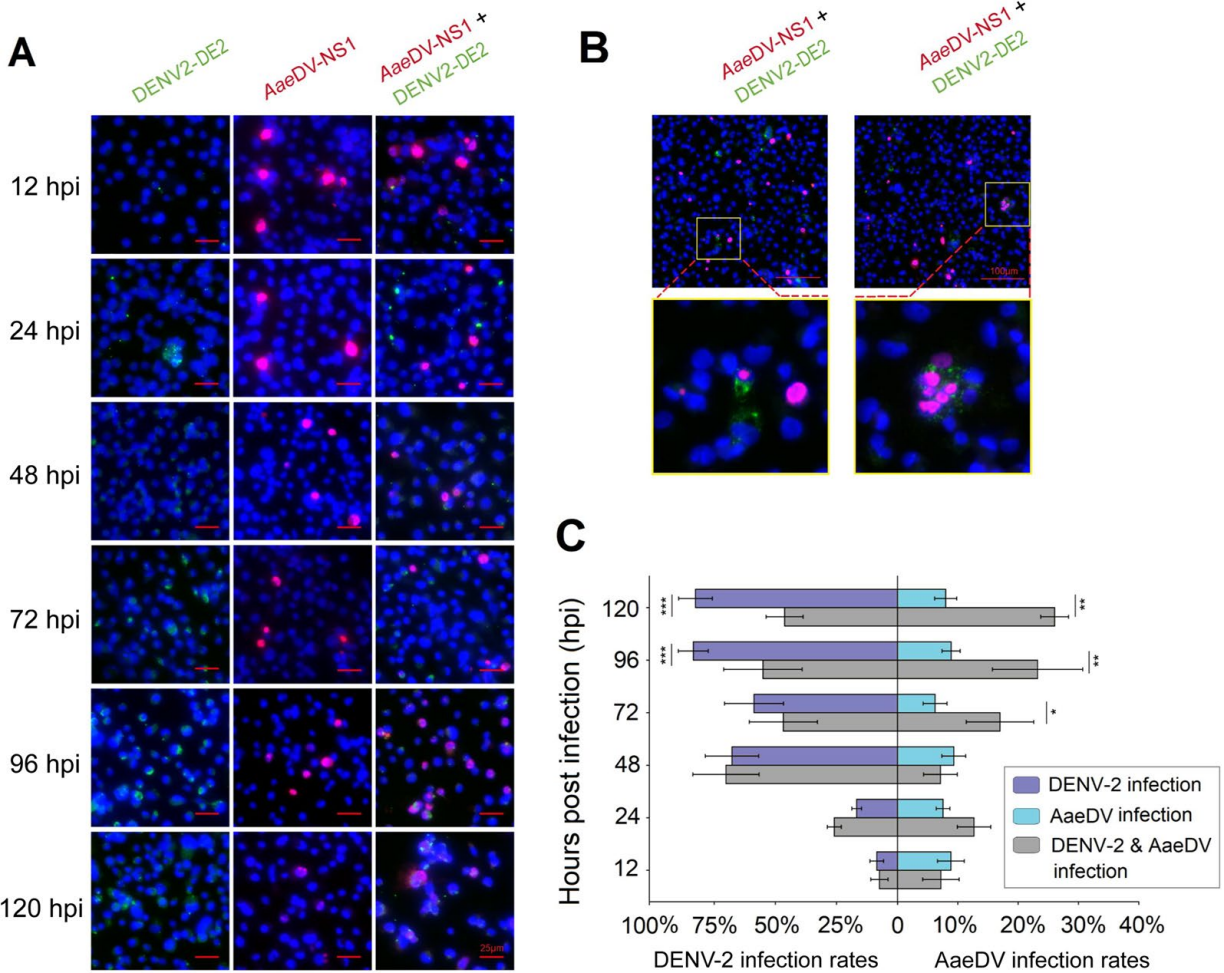
Immunofluorescence imaging of C6/36 cells monoinfected or superinfected with AaeDV and DENV-2. **A** C6/36 cells were subjected to AaeDV infection and then superinfected with DENV-2 for 12, 24, 48, 72, 96, and 120 h. AaeDV and DENV-2 monoinfection treatments were used as controls. The cells were fixed and permeabilized with an acetone/absolute alcohol mixture at the respective time points post infection and immunostained for DENV (green, AF488) and AaeDV (red, AF594). Images were captured under a microscope. DAPI was used to stain the nucleus (blue). **B** The top-right insets show a zoomed image of cells harboring both viruses. **C** A bar chart presents the percentage of C6/36 cells infected with AaeDV and DENV in monoinfection compared to superinfection. The given values are the mean ± SD of three independent replicates. *, *P* < 0.05; **, *P* < 0.01; ***, *P* < 0.001

The specific intracellular fluorescent signal for the DENV E2 envelope glycoprotein in C6/36 cells was decreased in DENV-2 E2 envelope glycoprotein-positive cells of approximately 38% and 63% at 96 hpi and 120 hpi, respectively, in comparison with the monoinfection control. However, a significantly increased rate of cells with AaeDV NS1-specific signals was observed in the superinfection treatment (72 hpi, *P* = 0.015; 96 hpi, *P* = 0.007; 120 hpi, *P* = 0.001), which suggests that DENV-2 infection indeed promoted replication of AaeDV in C6/36 cells (Fig. 4A and C). Moreover, confocal imaging showed the presence of both DENV-2 and AaeDV proteins in the same cell in less than 2% of cells at 96 hpi and 120 hpi (Fig. 4B).

### AaeDV infection affects the vector susceptibility DENV-2 in *Ae. albopictus*

**Fig. 5:**
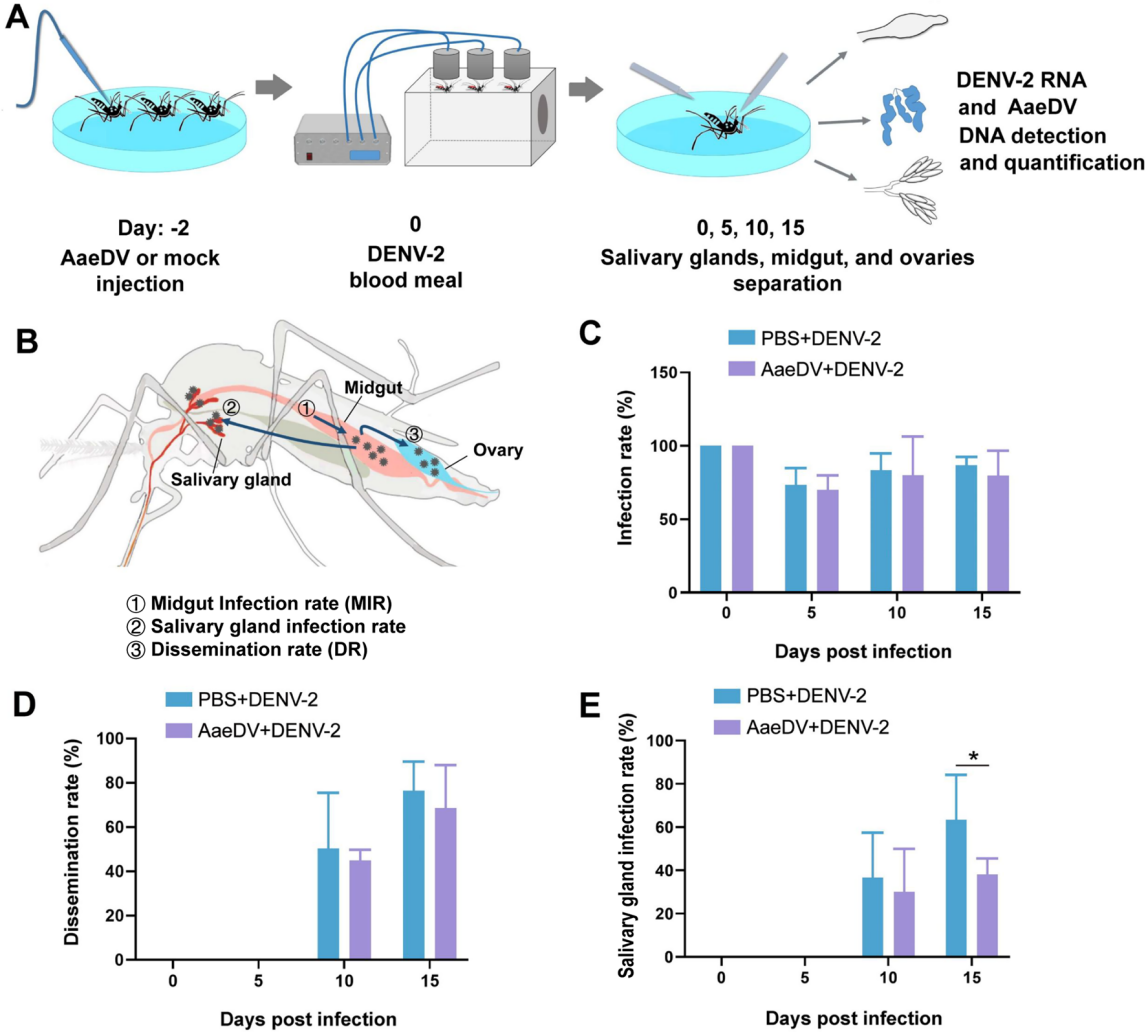
Vector susceptibility of *Ae. albopictus* orally infected with DENV-2 in monoinfected or superinfected adult females. **A** Diagram of the workflow used for detection of DENV-2 in different tissues of *Ae. albopictus*. The midguts, ovaries, and salivary glands of mosquitoes in monoinfected or superinfected were dissected at 0, 5, 10, and 15 days post infection and detected by PCR. **B** Schematic representation of the events that occur following ingestion of DENV-2-infected blood meal to subsequent transmission. The virus infects midgut epithelial cells by crossing the midgut infection barrier; the virus then disseminates to all organs, including the ovary and salivary glands. Infection of salivary glands is established by crossing the salivary gland infection barrier (SGIBs). Midgut infection rate (MIR) = the number of infected midguts/the number of tested midguts. Dissemination rate (DR) = the number of infected ovaries/the number of infected positive midguts. Salivary gland infection rate (SGIR) = the number of infected salivary glands/the total number of tested mosquitoes. **C** DENV-2 MIR in monoinfected or superinfected mosquitoes. **D** DENV-2 DR in monoinfected or superinfected mosquitoes. **E** DENV-2 SGIR in monoinfected or superinfected mosquitoes. Results are from three independent experiments (*n* = 29–30 per group), and error bars indicate the standard deviation

Only engorged females were used for dissection of the midgut, salivary glands, and ovary tissues at 0, 5, 10, and 15 dpi after exposure to DENV-2. Up to 15 dpi, adult mosquitoes injected with AaeDV did not show a significant increase in mortality. At 0 dpi, the IRs of mosquitoes were 100% in the superinfection and monoinfection treatments, indicating that all mosquitoes ingested the blood meal containing DENV-2 (Fig. 5C). After the infectious blood meal was digested, the IRs of *Ae. albopictus* were significantly reduced at 5 dpi compared with 0 dpi (z = 10.329, *P* = 0.001; z = 4.149, *P* = 0.0042; z = 4.565, *P* = 0.003) and then gradually increased at 10 hpi and 15 dpi (Fig. 5C). However, there was no significant difference in IR between superinfected and monoinfected mosquitoes at any time point (5 dpi, *P* = 0.774; 10 dpi, *P* = 0.739; 15 dpi, *P* = 0.685) (Fig. 5C). In superinfected mosquitos, DENV-2 was disseminated to the ovaries as early as in the monoinfected control, at 10 dpi. The DRs tended to decrease slightly at 10 dpi and 15 dpi; however, the change was not significant (10 dpi, *P* = 0.442; 15 dpi, *P* = 0.397) (Fig. 5D).

DENV-2 in the salivary glands was first detected at 10 dpi in both monoinfected and superinfected adult females, and the SGIR increased gradually over time (Fig. 5E). At 15 dpi, the SGIR in the superinfection treatment was significantly decreased by nearly 0.6-fold compared to that in the monoinfected control (*P* = 0.045) (Fig. 5E).

### Superinfection affects the amount of DENV-2 in *Ae. albopictus*

**Fig. 6:**
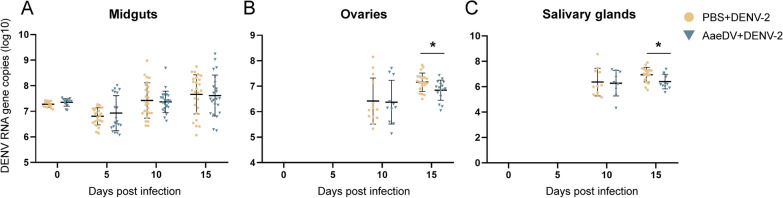
DENV-2 genome copy numbers in different tissues of monoinfected or superinfected* Ae. albopictus* adult females. DENV-2 RNA copy numbers in positive tissues of *Ae.* albopictus adults was quantified by qRT‒PCR. **A** Midguts; **B** ovaries; **C** salivary glands. Horizontal black lines indicate the mean value of DENV-2 RNA copy numbers (log10), and error bars indicate the standard deviation

The amounts of DENV-2 in the midguts, ovaries, and salivary glands of *Ae. albopictus* were further measured by qRT-PCR. AaeDV was also assessed by qPCR in the midgut, ovaries, and salivary gland of all samples to confirm infection (Fig. 6 and Additional file 6: Fig. S3).

In both monoinfection and superinfection treatments, DENV-2 copies (log_10_) in midguts decreased markedly from 0 (7.28 ± 0.12 and 7.35 ± 0.15) to 5 dpi (6.81 ± 0.34 and 6.93 ± 0.69) and then increased slowly over time. The level of DENV-2 peaked at 15 dpi (7.66 ± 0.77 and 7.62 ± 0.80). However, the DENV-2 RNA copies showed no significant differences between monoinfected or superinfected adult females at all time points examined (0 dpi, *P* = 0.177; 5 dpi, *P* = 0.448; 10 dpi, *P* = 0.743; 15 dpi, *P* = 0.835) (Fig. 6A).

In the ovaries, viral titration of monoinfection and superinfection treatments increased from 10 dpi (6.42 ± 0.90 and 6.37 ± 0.86) to 15 dpi (7.16 ± 0.36 and 6.84 ± 0.39), respectively (Fig. 6B). Notably, DENV-2 RNA copies in the superinfection treatments were significantly decreased compared to those in the monoinfection treatments at 15 dpi (*P* = 0.016) (Fig. 6B).

The trend of variation in the DENV-2 RNA copies in the salivary glands was similar to that in the ovaries (Fig. 6C). DENV-2 RNA copies in the salivary glands were higher at 15 dpi than at 10 dpi in monoinfection treatments, whereas DENV-2 copies in superinfection treatments showed no significant change between the two time points (*P* = 0.708). Furthermore, DENV-2 RNA copies in the superinfection treatments were significantly suppressed compared to those in the monoinfection treatments at 15 dpi (*P* = 0.020) (Fig. 6C).

## Discussion

Insect-specific viruses (ISVs) are a diverse group that exclusively infect insect hosts and do not infect vertebrates [33]. Most mosquito ISVs belong to Flaviviridae, whereas others are included in the families *Peribunyaviridae, Reoviridae, Birnaviridae,* and other taxa [34], and they play a crucial role in the mosquito microbiome due to their wide distribution and mosquito host range [35–38]. Mosquito-borne viruses (MBVs) are arboviruses that can be transmitted between mosquitoes and vertebrates, causing human diseases such as Zika virus (ZIKV), dengue virus (DENV), chikungunya virus (CHIKV), and Mayaro viruses (MAYV), leading to significant public health problems and economic burdens worldwide [39]. Since ISVs and MBVs both infect and replicate in mosquito vectors, the microbial community interaction between the ISVs and MBVs in mosquitoes is inevitable. The influence of ISVs on MBVs, even on the vector competence of mosquitoes, is undoubtedly becoming a major research topic in host–pathogen–vector interactions [40, 41].

The prevalent patterns of multiple infections of ISVs and MBVs fall into two broad categories: coinfection and superinfection. However, whether coinfection or superinfection occurs, and what sequential infection mode of superinfection is most frequent in natural mosquito populations remains unclear. In fact, it may be based on the vial geographical distribution, vector population infection rate, ecological factors, and other related epidemiological characteristics of the MBV host.

Guangdong is a high-risk area for dengue in China. Outbreaks of dengue were reported almost annually in Guangdong before the COVID-19 global pandemic, especially in Guangzhou, accounting for more than half of the DENV cases in Chinese mainland. Some studies have demonstrated that local *Ae. albopictus* mosquitoes are competent vectors for DENV [42–44], theoretically maintaining autochthonous dengue outbreaks in Guangdong Province. Few studies have reported DENV-positive *Ae. albopictus* detection by RT-PCR during the dengue epidemic in Guangzhou [45]. Moreover, no DENV was isolated from mosquitoes in the Guangzhou epidemic area. Evidence to date also supports the theory that dengue has not yet developed into an endemic arboviral disease in Guangdong.

For ISVs and MBVs, increasing evidence is emerging that interactions between ISVs and MBVs and complex viral communities have a significant impact on vector biology and even potential vector competence in MBV maintenance and transmission. Coinfection and superinfection of ISVs and MBVs have been studied in vitro and in vivo for many years, and interaction relations have also been observed between certain ISVs and MBVs. Although ISVs are widely distributed and persist in natural mosquito populations, MBVs have not yet been detected to significantly persist in wild mosquito populations in some regions, such as in Guangdong. Therefore, current research focuses more on MBV superinfection in ISV-persistent cells and mosquitoes. For example, *Anopheles gambiae* densovirus (AgDV) has a negative impact on Mayaro virus infection in both *An. gambiae* cells and mosquitoes [27]. Cell-fusing agent virus (CFAV) reduces DENV-1 and ZIKV dissemination in *Ae. aegypti* [46]. Persistent *Culex* flavivirus (CxFV) can inhibit WNV replication in *Cx. pipiens* [47] and also suppress the titer of DENV-1 and ZIKV in the head tissues of *Ae. aegypti* [46], leading to decreased MBV dissemination in mosquitoes. Espirito Santo virus (ESV) superinfection with DENV suppresses DENV-2 replication and hinders the vector competence of *Ae. aegypti* for DENV-2 [48]. The presence of Phasi Charoen-like virus (PCLV) leads to interference with ZIKV growth in *Ae. aegypti* Aag2 cells [49]. Infection with an insect-specific flavivirus, Nhumirim virus (NHUV), was demonstrated to restrict infection and transmission of ZIKV in *Ae. aegypti* [50]. An insect-specific alphavirus, Eilat virus (EILV), displays homologous and heterologous interference against Sindbis virus (SINV), Venezuelan equine encephalitis virus (VEEV), Eastern equine encephalitis virus (EEEV), Western equine encephalitis virus (WEEV), and CHIKV in *Ae. albopictus* C7/10 cells and delays dissemination of CHIKV in *Ae. aegypti* [51].

MDVs are mosquito-specific parvoviruses. As a kind of ISV with a very narrow host range, the prevalence of MDVs in natural populations still needs to be widely explored, especially in MBV-endemic regions, which will lay the foundation for further heterologous interference analysis that is closer to actual environmental situations. Our study provides a brief overview of the MDVs in field mosquitoes of China by NGS data analysis, and the results showed that MDVs are relatively widely distributed in wild mosquito populations of five PLADs, including *Ae. albopictus, Cx. pipiens, Cx. tritaeniorhyncus*, and *An. siniens.* We then focused on further detecting MDV in *Ae. albopictus* populations in Guangzhou by the traditional PCR method, and the results indicated an unexpectedly high rate of MDV positivity in the wild population in Guangzhou. Indeed, the MDV positive rate in populations of all three different habitats exceeded 65%, higher than previously reported [52]. Therefore, our research focused on DENV superinfection in *Ae. albopictus* cells and adults with persisting MDVs. In vitro, AaeDV-persistent C6/36 cells were superinfected with DENV-2, and gene copy numbers were quantified both in cell pellets and supernatants at different time points. For DENV-2, although no significant difference was observed at most time points, a decreased DENV-2 RNA level in DENV-2 monoinfection cells was observed at 120 hpi. In the superinfection treatment, the DENV-2 RNA copies were increased in cells but decreased in the supernatant. Hence, we further determined the percentage of virus-positive cells by IFA, and DENV-2 E2 envelope glycoprotein-positive cells were relatively decreased by approximately 38% and 63% at 96 hpi and 120 hpi, respectively. The mechanisms of this phenomenon remain unknown. We cannot exclude that DENV-2 E2 envelope glycoprotein-negative cells were not infected, considering the possibility that they only contained viral genomic material, but the results indicated that AaeDV infection may not have significantly affected DENV-2 replication in cells but rather influenced translation of DENV-2, causing a decrease in the DENV copies in the supernatant and DENV-2 E2 envelope glycoprotein-positive cell percentage. Considering in vivo studies that closely recapitulate natural MBV transmission settings, we further determined the impact of AaeDV infection on the vector competence of *Ae. albopictus* adults for DENV-2. The SIE was obviously observed in vivo, and the salivary gland infection rate was significantly decreased in superinfected mosquitoes. However, the salivary gland infection rate does not necessarily reflect the changes in transmission since the virus in salivary glands does not directly correlate with that in saliva [53]. Moreover, the reduction of SIG and DENV-2 genome copy numbers ovaries and salivary glands, indicated the impaired susceptibility and vector competence to DENV-2 in *Ae. albopictus*. Further evidence is needed to determine whether this reduction in DENV-2 replication in mosquito results in a decrease in its transmission.

Our findings provide evidence that MDV heterologous interference affects mosquito vector competence for DENV. Further investigations are necessary to determine whether the reduction in DENV-2 replication and positive rate in salivary glands will ultimately lead to a decreased DENV-2 transmission. Additionally, it is crucial to clarify the underlying mechanism and reconcile the consistent and contradictory observations provided by different research teams [54–57], which may reflect the large variety of cell lines, mosquito species, geographic strain, genetic background, virus strains, and experimental methods. Ultimately, these data demonstrate the complex interactions that occur when MDV and DENV share the same vector host, enhance proposed strategies for the development of MDVs as biocontrol agents against MBVs and indicate a potential novel tool for mosquito-transmitted disease control.

Although the present study provides evidence that AaeDV infection can impact the susceptibility of mosquito vectors to DENV-2 both in vitro and in vivo. However, it is important to acknowledge that the obtained results may be limited by the particular mosquito population and virus isolate utilized in the experiments.

## Conclusions

MDV is widely present in natural mosquito populations, and replication of DENV-2 is suppressed in MDV-infected *Ae. albopictus*, thus reducing vector susceptibility to DENV-2. Our study supports the hypothesis that MDV may contribute to reducing transmission of DENV and provides an alternative strategy for mosquito-transmitted disease control.

## Supplementary Information

**Additional file 1**: **Table S1**. Illumina sequencing datasets of field-caught mosquitoes used in prevalence analysis of mosquito densovirus.

**Additional file 2**: **Table S2**. Accession number of the mosquito densovirus reference.

**Additional file 3**: **Table S3**. Primers used in the study.

**Additional file 4**: **Figure S1**. Mosquito densovirus infection in natural mosquito populations. The mosquito densovirus positive rates of reads of in field mosquito metagenomic data from five provincial level administrative divisions. NX, Ningxia Hui Autonomous Region; SX, Shanxi Province; GS, Gansu Province; YN, Yunnan Province; GD, Guangdong Province. MDV positive rate in natural mosquito populations in Guangzhou. The Guangzhou region is labeled in light yellow on the map of China. The zoomed inset shows a magnification of the area of Guangzhou, and different colors indicate the different districts in Guangzhou. The dots indicate the locations of mosquito collection sites. The pie charts in the figure show the MDV positive rate in natural mosquito populations at each collection site. The base layer of this modified map originated from National Earth System Science Data Center, National Science & Technology Infrastructure of China.

**Additional file 5**: **Figure S2**. Detection of MDV positive rates in natural mosquito populations in Guangzhou.

**Additional file 6**: **Figure S3**. AaeDV DNA copies in midguts, ovaries, and salivary gland of AaeDV-infected Aedes albopictus at different days post infection.

## Data Availability

The data used in this study are available, if necessary; please contact the first author (LK) or corresponding author (JBG).

## Abbreviations

DENV: Dengue virus
MDV: Mosquito densovirus
AaeDV: *Aedes aegypti* densovirus
TCID_50_: Median tissue culture infective dose
a.s.l.: Above sea level
PBS: Phosphate-buffered saline
IFA: Indirect immunofluorescence assay
FBS: Fetal bovine serum
RT‒PCR: Reverse transcription polymerase chain reaction
RT‒qPCR: Quantitative reverse transcription polymerase chain reaction
RNA: Ribonucleic acid
cDNA: Complementary deoxyribonucleic acid
NS1: Nonstructural protein 1
RPMI: Roswell Park Memorial Institute
ISV: Insect-specific viruses
