# Supplementary material for: Mosquito densovirus significantly reduces the vector susceptibility to dengue virus serotype 2 in Aedes albopictus mosquitoes (Diptera: Culicidae)
Source: Infect Dis Poverty. 2023 May 9;12:48. doi: 10.1186/s40249-023-01099-8 (PMC10169196; doi:10.1186/s40249-023-01099-8)
Supplement: Supplementary file 3 — Additional file 3: Table S3. Primers used in the study. [file 40249_2023_1099_MOESM3_ESM.docx]

| **Table S3. Primers used in the study** | | | |
| --- | --- | --- | --- |
| **Primer name** | **Sequences （5’-3’）** | **Annealing temperature and cycles** | **Usage of primer** |
| MDV899-F  MDV1554-R | ATAACGGGTCACAGGCAAGCA  GTTTCGATACCGTAACGGATGC | 55 °C, 35 | MDV detection in natural mosquito populations |
| *Rps7*-F  *Rps7*-R | TGATGCGTTCGAGGGTCAAA  TCTGGAAGGCCTTCTGCTTG | 55 °C, 35 | Internal control amplification |
| DENV2-F  DENV2-R | CACGAGAACCCAAGAACCGA  TGGTCTTTCCCAGCGTCAAT | 55 °C, 35 | PCR products was cloned into pMD18-T |
| DENV2-qF  DENV2-qR | ACAAGTCGAACAACCTGGTCCAT  GCCGCACCATTGGTCTTCTC | 55 °C, 40 | DENV-2 RNA copy numbers determination with qPCR |
| AaeDV-qF  AaeDV-qR | CAGGAGGAAACAGCACAAGA  GTTTCGATACCGTAACGGATGC | 55 °C, 40 | AaeDV genome copy numbers determination with qPCR |
