# Supplementary figures and images for: Mosquito densovirus significantly reduces the vector susceptibility to dengue virus serotype 2 in Aedes albopictus mosquitoes (Diptera: Culicidae)
Source: Infect Dis Poverty. 2023 May 9;12:48. doi: 10.1186/s40249-023-01099-8 (PMC10169196; doi:10.1186/s40249-023-01099-8)

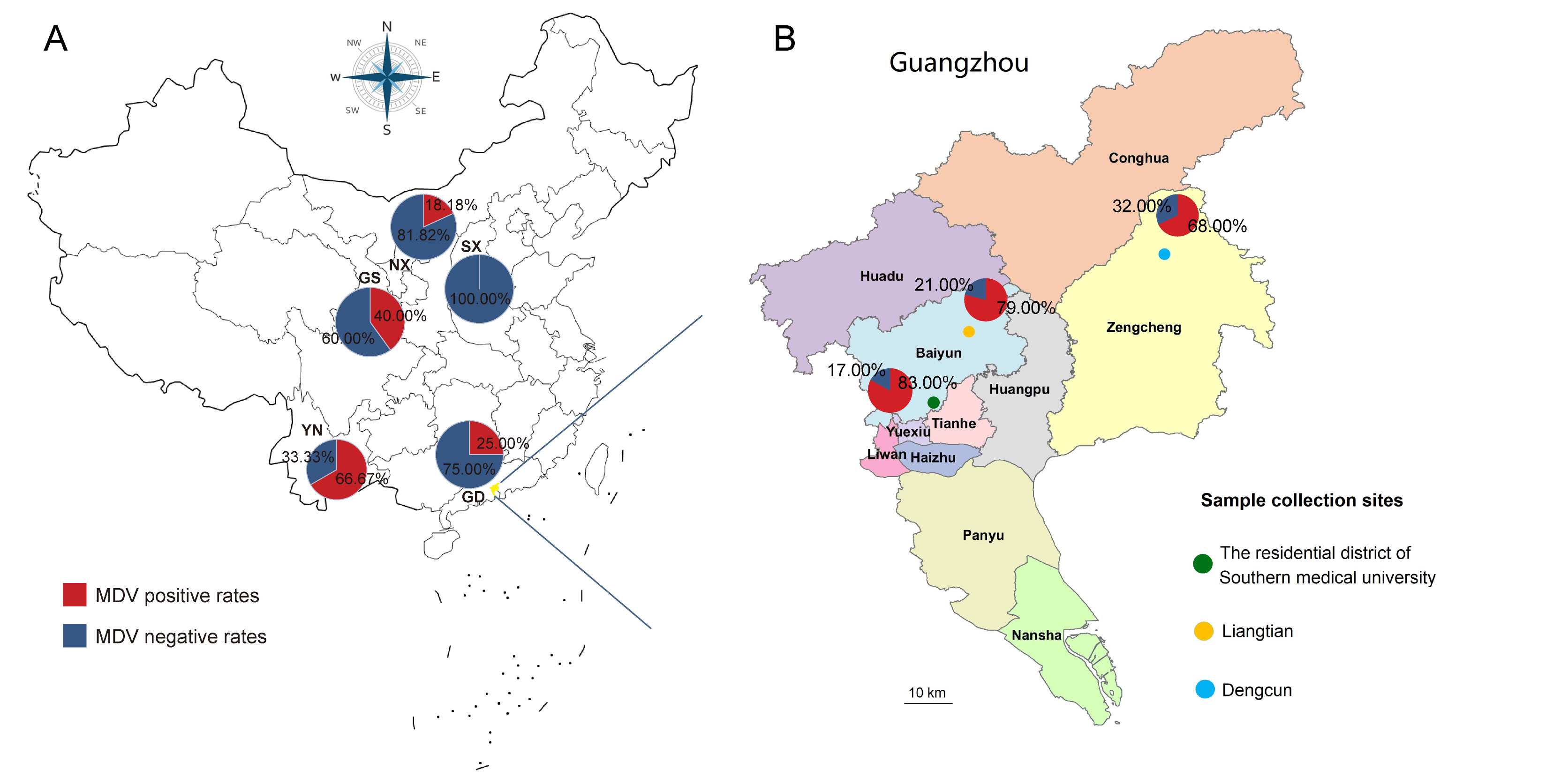

Supplement: Supplementary file 4 — Additional file 4: Figure S1. Mosquito densovirus infection in natural mosquito populations. The mosquito densovirus positive rates of reads of in field mosquito metagenomic data from five provincial level administrative divisions. NX, Ningxia Hui Autonomous Region; SX, Shanxi Province; GS, Gansu Province; YN, Yunnan Province; GD, Guangdong Province. MDV positive rate in natural mosquito populations in Guangzhou. The Guangzhou region is labeled in light yellow on the map of China. The zoomed inset shows a magnification of the area of Guangzhou, and different colors indicate the different districts in Guangzhou. The dots indicate the locations of mosquito collection sites. The pie charts in the figure show the MDV positive rate in natural mosquito populations at each collection site. The base layer of this modified map originated from National Earth System Science Data Center, National Science & Technology Infrastructure of China. [file 40249_2023_1099_MOESM4_ESM.tif]

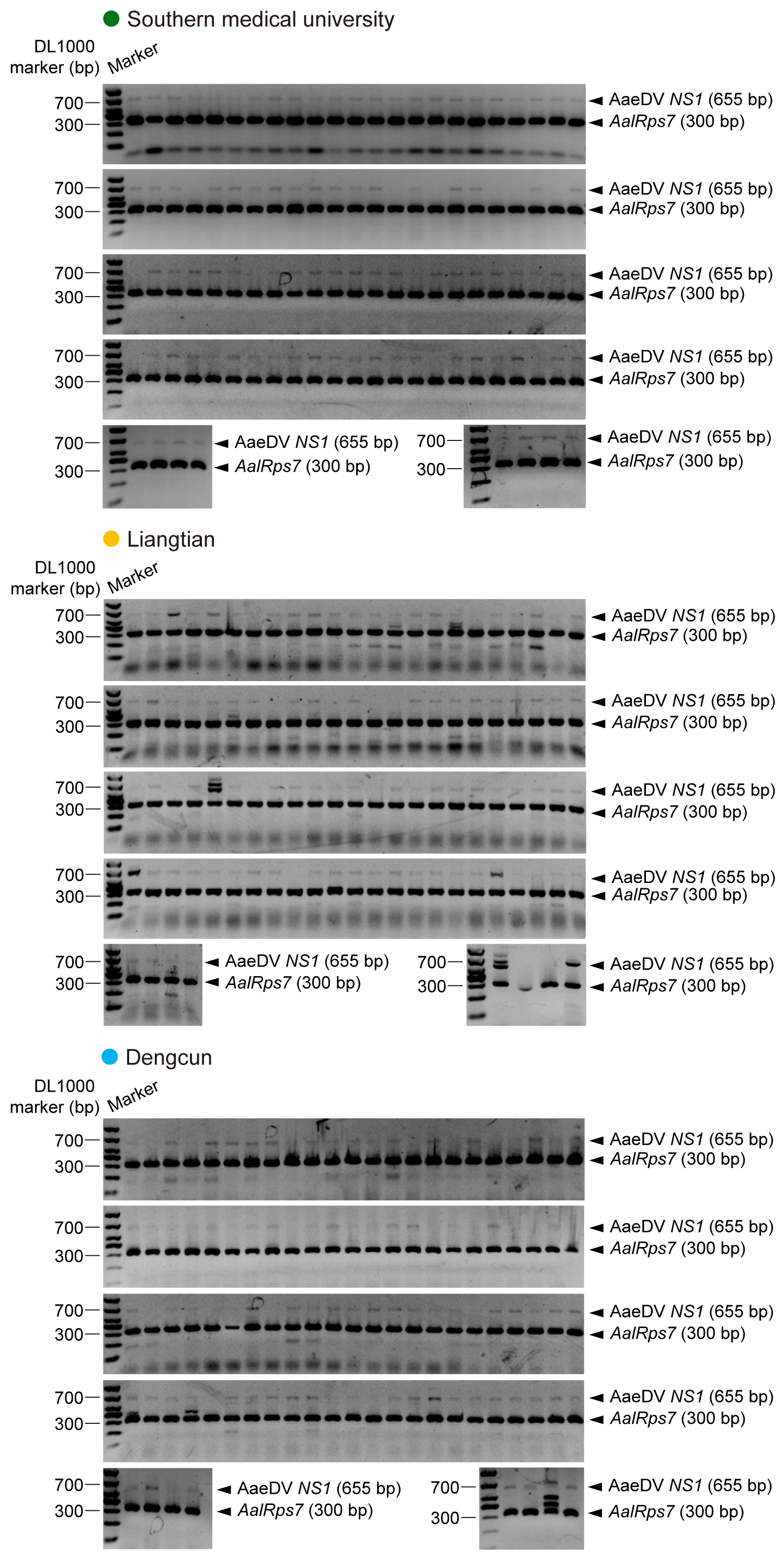

Supplement: Supplementary file 5 — Additional file 5: Figure S2. Detection of MDV positive rates in natural mosquito populations in Guangzhou. [file 40249_2023_1099_MOESM5_ESM.tif]

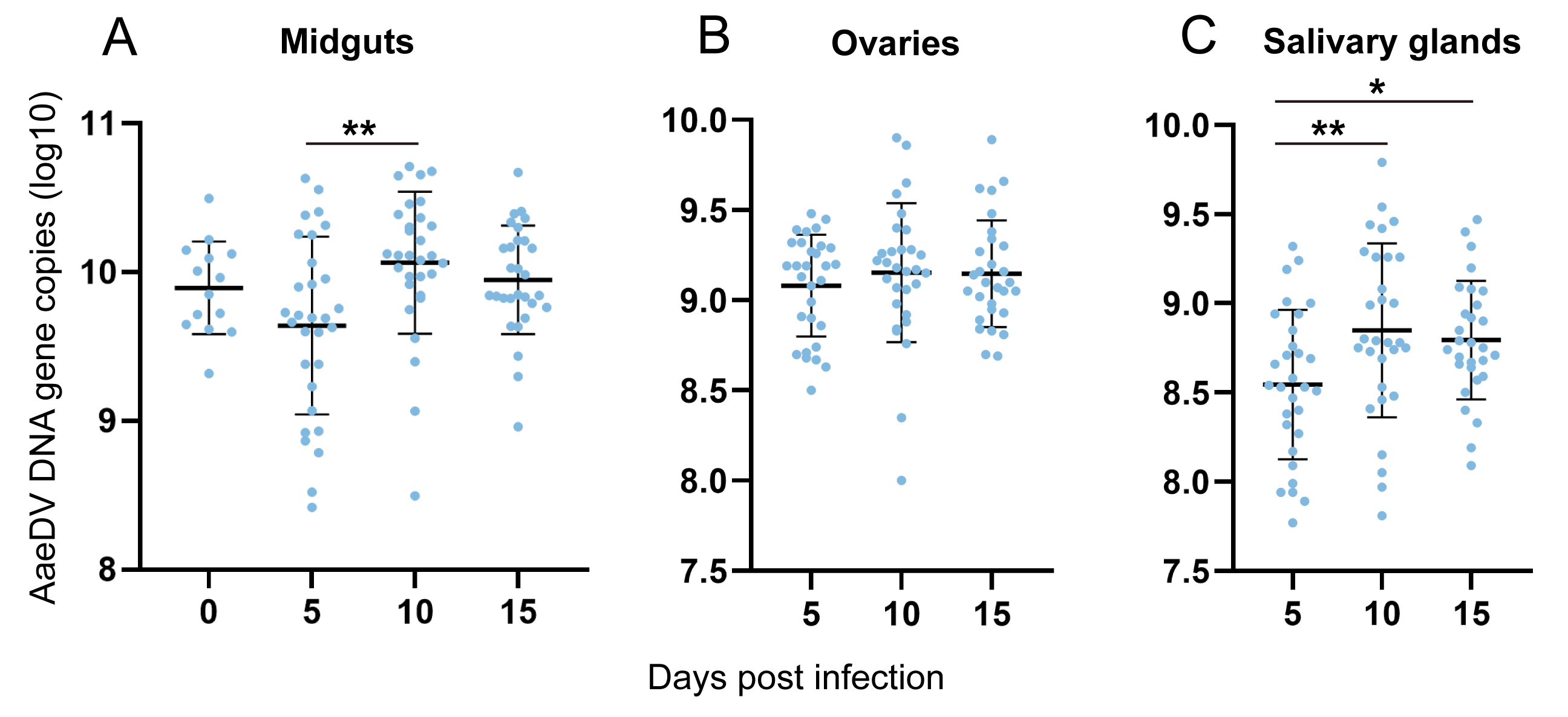

Supplement: Supplementary file 6 — Additional file 6: Figure S3. AaeDV DNA copies in midguts, ovaries, and salivary gland of AaeDV-infected Aedes albopictus at different days post infection. [file 40249_2023_1099_MOESM6_ESM.tif]
